# 2-Chloro-*N*-(4-chloro-3-iodo­phen­yl)-4-(methyl­sulfon­yl)benzamide

**DOI:** 10.1107/S1600536811053633

**Published:** 2011-12-21

**Authors:** Dao-Cai Wang, Hang Song, Chang Yang, Wen-Cai Huang, Shun Yao

**Affiliations:** a Department of Pharmaceutical and Biological Engineering, College of Chemical Engineering, Sichuan University, Chengdu 610065, People’s Republic of China

## Abstract

In the title compound, C_14_H_10_Cl_2_INO_3_S, the dihedral angle between the benzene rings is 52.13 (10)°. In the crystal, the components are linked by pairs of N—H⋯O(sulfon­yl) hydrogen bonds into centrosymmetric dimers.

## Related literature

For background to benzamides, see: Castanedo *et al.* (2010 ▶); Tremblay *et al.* (2010 ▶); Mahindroo *et al.* (2010 ▶). For the preparation of the title compound, see: Robarge *et al.* (2009 ▶).
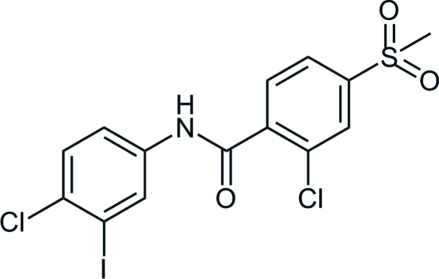


## Experimental

### 

#### Crystal data

C_14_H_10_Cl_2_INO_3_S
*M* *_r_* = 470.09
Triclinic, 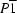

*a* = 8.8694 (6) Å
*b* = 10.3837 (8) Å
*c* = 10.4288 (5) Å
α = 103.862 (5)°
β = 96.452 (5)°
γ = 114.949 (7)°
*V* = 820.25 (9) Å^3^
*Z* = 2
Mo *K*α radiation
μ = 2.41 mm^−1^
*T* = 290 K
0.30 × 0.30 × 0.25 mm

#### Data collection

Oxford Diffraction Xcalibur Eos diffractometer
Absorption correction: multi-scan (*CrysAlis PRO*; Oxford Diffraction, 2006 ▶) *T*_min_ = 0.887, *T*_max_ = 1.000
6770 measured reflections
3323 independent reflections
2957 reflections with *I* > 2σ(*I*)
*R*_int_ = 0.020

#### Refinement

*R*[*F* ^2^ > 2σ(*F* ^2^)] = 0.028
*wR*(*F* ^2^) = 0.067
*S* = 1.07
3323 reflections
200 parameters
H-atom parameters constrained
Δρ_max_ = 0.43 e Å^−3^
Δρ_min_ = −0.55 e Å^−3^

### 

Data collection: *CrysAlis PRO* (Oxford Diffraction, 2006 ▶); cell refinement: *CrysAlis PRO*; data reduction: *CrysAlis PRO*; program(s) used to solve structure: *SHELXS97* (Sheldrick, 2008 ▶); program(s) used to refine structure: *SHELXL97* (Sheldrick, 2008 ▶); molecular graphics: *OLEX2* (Dolomanov *et al.*, 2009 ▶) and *Mercury* (Macrae *et al.*, 2006 ▶); software used to prepare material for publication: *OLEX2*.

## Supplementary Material

Crystal structure: contains datablock(s) I, global. DOI: 10.1107/S1600536811053633/tk5034sup1.cif

Structure factors: contains datablock(s) I. DOI: 10.1107/S1600536811053633/tk5034Isup2.hkl

Supplementary material file. DOI: 10.1107/S1600536811053633/tk5034Isup3.cml

Additional supplementary materials:  crystallographic information; 3D view; checkCIF report

## Figures and Tables

**Table 1:** Hydrogen-bond geometry (Å, °)

*D*—H⋯*A*	*D*—H	H⋯*A*	*D*⋯*A*	*D*—H⋯*A*
N1—H1⋯O2^i^	0.86	2.15	2.991 (3)	167

Symmetry code: (i) 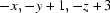.

**Fig. 1.**
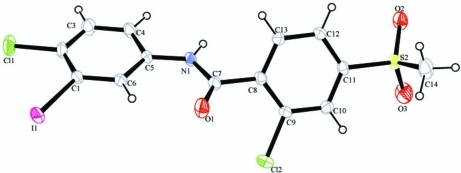
The molecular structure of the title compound, with displacement ellipsoids drawn at the 30% probability level.

## supplementary crystallographic information

### Comment

The benzamide moiety is the constituent of many biologically significant compounds including anticancer compounds (Castanedo *et al.*, 2010; Tremblay *et al.*, 2010; Mahindroo *et al.*, 2010). The title compound is one of the key intermediates in our synthetic investigations of anticancer drugs. In this paper, we synthesized the title compound and report its crystal structure, Fig. 1. The dihedral angle between the two benzene rings is 52.13 (10)°. In the crystal, the molecules are connected *via* intermolecular N—H···O hydrogen bonds to form a centrosymmetric dimer, in which the amide H atom acts as a donor and the sulfonyl-O atom act as an acceptor, Table 1.

### Experimental

The title compound was prepared by a method similar to that of Robarge *et al.* (2009). Crystals were obtained by slow evaporation from its methanol-acetic acid (5:2) solution.

### Refinement

All H atoms were positioned geometrically (C—H = 0.93–0.96 Å; N—H = 0.86 Å) and allowed to ride on their parent atoms, with *U*_iso_(H) = 1.2*U*_eq_(parent).

### Crystal data

**Table:**

C_14_H_10_Cl_2_INO_3_S	*Z* = 2
*M**_r_* = 470.09	*F*(000) = 456
Triclinic, *P*1	*D*_x_ = 1.903 Mg m^−^^3^
Hall symbol: -P 1	Mo *K*α radiation, λ = 0.7107 Å
*a* = 8.8694 (6) Å	Cell parameters from 2872 reflections
*b* = 10.3837 (8) Å	θ = 2.9–26.3°
*c* = 10.4288 (5) Å	µ = 2.41 mm^−^^1^
α = 103.862 (5)°	*T* = 290 K
β = 96.452 (5)°	Block, colourless
γ = 114.949 (7)°	0.30 × 0.30 × 0.25 mm
*V* = 820.25 (9) Å^3^	

### Data collection

**Table:**

Oxford Diffraction Xcalibur Eos diffractometer	3323 independent reflections
Radiation source: Enhance (Mo) X-ray Source	2957 reflections with *I* > 2σ(*I*)
graphite	*R*_int_ = 0.020
Detector resolution: 16.0874 pixels mm^-1^	θ_max_ = 26.4°, θ_min_ = 2.9°
ω scans	*h* = −11→11
Absorption correction: multi-scan (*CrysAlis PRO*; Oxford Diffraction, 2006)	*k* = −12→12
*T*_min_ = 0.887, *T*_max_ = 1.000	*l* = −12→13
6770 measured reflections	

### Refinement

**Table:**

Refinement on *F*^2^	Primary atom site location: structure-invariant direct methods
Least-squares matrix: full	Secondary atom site location: difference Fourier map
*R*[*F*^2^ > 2σ(*F*^2^)] = 0.028	Hydrogen site location: inferred from neighbouring sites
*wR*(*F*^2^) = 0.067	H-atom parameters constrained
*S* = 1.07	*w* = 1/[σ^2^(*F*_o_^2^) + (0.0257*P*)^2^ + 0.4057*P*] where *P* = (*F*_o_^2^ + 2*F*_c_^2^)/3
3323 reflections	(Δ/σ)_max_ = 0.001
200 parameters	Δρ_max_ = 0.43 e Å^−^^3^
0 restraints	Δρ_min_ = −0.55 e Å^−^^3^

### Special details

**Table:**

Geometry. All e.s.d.'s (except the e.s.d. in the dihedral angle between two l.s. planes) are estimated using the full covariance matrix. The cell e.s.d.'s are taken into account individually in the estimation of e.s.d.'s in distances, angles and torsion angles; correlations between e.s.d.'s in cell parameters are only used when they are defined by crystal symmetry. An approximate (isotropic) treatment of cell e.s.d.'s is used for estimating e.s.d.'s involving l.s. planes.
Refinement. Refinement of *F*^2^ against ALL reflections. The weighted *R*-factor *wR* and goodness of fit *S* are based on *F*^2^, conventional *R*-factors *R* are based on *F*, with *F* set to zero for negative *F*^2^. The threshold expression of *F*^2^ > σ(*F*^2^) is used only for calculating *R*-factors(gt) *etc*. and is not relevant to the choice of reflections for refinement. *R*-factors based on *F*^2^ are statistically about twice as large as those based on *F*, and *R*- factors based on ALL data will be even larger.

### Fractional atomic coordinates and isotropic or equivalent isotropic displacement parameters (Å^2^)

**Table:**

	*x*	*y*	*z*	*U*_iso_*/*U*_eq_	
I1	0.61447 (3)	0.68561 (3)	0.77089 (2)	0.04648 (9)	
Cl1	0.29725 (14)	0.32782 (12)	0.59427 (9)	0.0627 (3)	
Cl2	0.29306 (15)	0.99624 (11)	1.34350 (10)	0.0603 (3)	
S2	0.11153 (10)	0.80692 (9)	1.76536 (8)	0.03759 (19)	
O1	0.4697 (3)	0.8156 (3)	1.2278 (2)	0.0620 (8)	
O2	0.0367 (3)	0.6656 (3)	1.7893 (2)	0.0536 (7)	
O3	0.0121 (4)	0.8840 (4)	1.7587 (3)	0.0676 (8)	
N1	0.2439 (3)	0.5816 (3)	1.1495 (2)	0.0344 (6)	
H1	0.1576	0.5214	1.1721	0.041*	
C1	0.4083 (4)	0.5502 (4)	0.8367 (3)	0.0328 (7)	
C2	0.2898 (4)	0.4068 (4)	0.7585 (3)	0.0390 (8)	
C3	0.1603 (4)	0.3219 (4)	0.8106 (3)	0.0478 (9)	
H3	0.0821	0.2240	0.7588	0.057*	
C4	0.1467 (4)	0.3817 (4)	0.9391 (3)	0.0412 (8)	
H4	0.0578	0.3246	0.9729	0.049*	
C5	0.2643 (4)	0.5261 (3)	1.0182 (3)	0.0303 (6)	
C6	0.3978 (4)	0.6101 (3)	0.9675 (3)	0.0326 (7)	
H6	0.4798	0.7061	1.0210	0.039*	
C7	0.3431 (4)	0.7173 (4)	1.2433 (3)	0.0370 (7)	
C8	0.2843 (4)	0.7407 (3)	1.3728 (3)	0.0315 (7)	
C9	0.2612 (4)	0.8646 (3)	1.4266 (3)	0.0346 (7)	
C10	0.2081 (4)	0.8860 (3)	1.5459 (3)	0.0346 (7)	
H10	0.1917	0.9691	1.5806	0.042*	
C11	0.1800 (3)	0.7808 (3)	1.6127 (3)	0.0307 (6)	
C12	0.2046 (4)	0.6571 (3)	1.5633 (3)	0.0332 (7)	
H12	0.1865	0.5880	1.6099	0.040*	
C13	0.2566 (4)	0.6382 (4)	1.4433 (3)	0.0336 (7)	
H13	0.2734	0.5553	1.4090	0.040*	
C14	0.3027 (5)	0.9225 (5)	1.8925 (3)	0.0601 (11)	
H14A	0.2762	0.9474	1.9789	0.090*	
H14B	0.3667	1.0126	1.8717	0.090*	
H14C	0.3697	0.8705	1.8963	0.090*	

### Atomic displacement parameters (Å^2^)

**Table:**

	*U*^11^	*U*^22^	*U*^33^	*U*^12^	*U*^13^	*U*^23^
I1	0.05672 (15)	0.05233 (16)	0.04928 (15)	0.02995 (12)	0.03477 (12)	0.02942 (12)
Cl1	0.0823 (7)	0.0685 (7)	0.0327 (4)	0.0349 (6)	0.0224 (5)	0.0051 (4)
Cl2	0.0951 (7)	0.0446 (5)	0.0564 (5)	0.0341 (5)	0.0333 (5)	0.0322 (4)
S2	0.0338 (4)	0.0384 (4)	0.0326 (4)	0.0090 (3)	0.0162 (3)	0.0088 (3)
O1	0.0644 (16)	0.0378 (14)	0.0517 (15)	−0.0044 (13)	0.0346 (13)	0.0034 (12)
O2	0.0539 (14)	0.0436 (15)	0.0454 (13)	0.0031 (12)	0.0266 (12)	0.0140 (11)
O3	0.0703 (18)	0.094 (2)	0.0684 (18)	0.0555 (18)	0.0411 (15)	0.0315 (16)
N1	0.0369 (13)	0.0297 (14)	0.0319 (13)	0.0082 (11)	0.0193 (11)	0.0108 (11)
C1	0.0395 (16)	0.0390 (18)	0.0308 (15)	0.0225 (14)	0.0159 (13)	0.0181 (14)
C2	0.0490 (18)	0.048 (2)	0.0240 (15)	0.0270 (17)	0.0120 (14)	0.0086 (14)
C3	0.0491 (19)	0.040 (2)	0.0351 (18)	0.0103 (17)	0.0068 (15)	0.0020 (15)
C4	0.0386 (16)	0.0398 (19)	0.0350 (17)	0.0075 (15)	0.0120 (14)	0.0134 (14)
C5	0.0358 (15)	0.0330 (16)	0.0258 (14)	0.0157 (13)	0.0129 (12)	0.0134 (12)
C6	0.0356 (15)	0.0292 (16)	0.0325 (15)	0.0128 (13)	0.0116 (13)	0.0117 (13)
C7	0.0403 (17)	0.0300 (17)	0.0345 (16)	0.0089 (14)	0.0156 (14)	0.0107 (14)
C8	0.0283 (14)	0.0296 (16)	0.0300 (15)	0.0065 (13)	0.0098 (12)	0.0104 (13)
C9	0.0421 (16)	0.0282 (16)	0.0324 (15)	0.0126 (14)	0.0123 (14)	0.0135 (13)
C10	0.0367 (16)	0.0297 (16)	0.0345 (16)	0.0132 (14)	0.0105 (13)	0.0083 (13)
C11	0.0267 (13)	0.0341 (17)	0.0260 (14)	0.0092 (13)	0.0078 (12)	0.0092 (12)
C12	0.0357 (15)	0.0314 (17)	0.0337 (15)	0.0135 (13)	0.0122 (13)	0.0147 (13)
C13	0.0390 (16)	0.0320 (17)	0.0332 (16)	0.0178 (14)	0.0142 (13)	0.0112 (13)
C14	0.056 (2)	0.051 (2)	0.0362 (19)	−0.0018 (19)	0.0054 (17)	0.0050 (17)

### Geometric parameters (Å, °)

**Table:**

I1—C1	2.090 (3)	C4—C5	1.382 (4)
Cl1—C2	1.735 (3)	C5—C6	1.391 (4)
Cl2—C9	1.731 (3)	C6—H6	0.9300
S2—O2	1.431 (3)	C7—C8	1.504 (4)
S2—O3	1.426 (3)	C8—C9	1.384 (4)
S2—C11	1.769 (3)	C8—C13	1.388 (4)
S2—C14	1.757 (4)	C9—C10	1.381 (4)
O1—C7	1.218 (4)	C10—H10	0.9300
N1—H1	0.8600	C10—C11	1.383 (4)
N1—C5	1.416 (4)	C11—C12	1.380 (4)
N1—C7	1.348 (4)	C12—H12	0.9300
C1—C2	1.375 (5)	C12—C13	1.381 (4)
C1—C6	1.388 (4)	C13—H13	0.9300
C2—C3	1.381 (5)	C14—H14A	0.9600
C3—H3	0.9300	C14—H14B	0.9600
C3—C4	1.378 (4)	C14—H14C	0.9600
C4—H4	0.9300		
			
O2—S2—C11	107.98 (15)	O1—C7—N1	124.5 (3)
O2—S2—C14	106.74 (19)	O1—C7—C8	121.2 (3)
O3—S2—O2	118.76 (18)	N1—C7—C8	114.3 (2)
O3—S2—C11	108.53 (16)	C9—C8—C7	121.8 (3)
O3—S2—C14	109.7 (2)	C9—C8—C13	118.5 (3)
C14—S2—C11	104.21 (16)	C13—C8—C7	119.7 (3)
C5—N1—H1	116.0	C8—C9—Cl2	120.7 (2)
C7—N1—H1	116.0	C10—C9—Cl2	117.7 (3)
C7—N1—C5	128.0 (2)	C10—C9—C8	121.6 (3)
C2—C1—I1	123.1 (2)	C9—C10—H10	120.8
C2—C1—C6	120.3 (3)	C9—C10—C11	118.4 (3)
C6—C1—I1	116.6 (2)	C11—C10—H10	120.8
C1—C2—Cl1	121.8 (2)	C10—C11—S2	118.7 (2)
C1—C2—C3	119.8 (3)	C12—C11—S2	119.6 (2)
C3—C2—Cl1	118.3 (3)	C12—C11—C10	121.7 (3)
C2—C3—H3	119.9	C11—C12—H12	120.7
C4—C3—C2	120.3 (3)	C11—C12—C13	118.6 (3)
C4—C3—H3	119.9	C13—C12—H12	120.7
C3—C4—H4	119.8	C8—C13—H13	119.4
C3—C4—C5	120.4 (3)	C12—C13—C8	121.3 (3)
C5—C4—H4	119.8	C12—C13—H13	119.4
C4—C5—N1	117.9 (3)	S2—C14—H14A	109.5
C4—C5—C6	119.4 (3)	S2—C14—H14B	109.5
C6—C5—N1	122.7 (3)	S2—C14—H14C	109.5
C1—C6—C5	119.8 (3)	H14A—C14—H14B	109.5
C1—C6—H6	120.1	H14A—C14—H14C	109.5
C5—C6—H6	120.1	H14B—C14—H14C	109.5
			
I1—C1—C2—Cl1	−2.5 (4)	C4—C5—C6—C1	2.2 (5)
I1—C1—C2—C3	177.7 (3)	C5—N1—C7—O1	0.1 (6)
I1—C1—C6—C5	−179.8 (2)	C5—N1—C7—C8	179.6 (3)
Cl1—C2—C3—C4	−178.1 (3)	C6—C1—C2—Cl1	179.6 (2)
Cl2—C9—C10—C11	179.2 (2)	C6—C1—C2—C3	−0.2 (5)
S2—C11—C12—C13	179.4 (2)	C7—N1—C5—C4	179.5 (3)
O1—C7—C8—C9	51.1 (5)	C7—N1—C5—C6	0.2 (5)
O1—C7—C8—C13	−127.0 (4)	C7—C8—C9—Cl2	2.0 (4)
O2—S2—C11—C10	160.6 (2)	C7—C8—C9—C10	−179.6 (3)
O2—S2—C11—C12	−19.6 (3)	C7—C8—C13—C12	179.2 (3)
O3—S2—C11—C10	30.7 (3)	C8—C9—C10—C11	0.8 (5)
O3—S2—C11—C12	−149.6 (3)	C9—C8—C13—C12	1.0 (4)
N1—C5—C6—C1	−178.5 (3)	C9—C10—C11—S2	−179.9 (2)
N1—C7—C8—C9	−128.4 (3)	C9—C10—C11—C12	0.4 (4)
N1—C7—C8—C13	53.5 (4)	C10—C11—C12—C13	−0.8 (4)
C1—C2—C3—C4	1.8 (6)	C11—C12—C13—C8	0.1 (4)
C2—C1—C6—C5	−1.7 (5)	C13—C8—C9—Cl2	−179.8 (2)
C2—C3—C4—C5	−1.3 (6)	C13—C8—C9—C10	−1.5 (4)
C3—C4—C5—N1	−180.0 (3)	C14—S2—C11—C10	−86.2 (3)
C3—C4—C5—C6	−0.7 (5)	C14—S2—C11—C12	93.6 (3)

### Hydrogen-bond geometry (Å, °)

**Table:**

*D*—H···*A*	*D*—H	H···*A*	*D*···*A*	*D*—H···*A*
N1—H1···O2^i^	0.86	2.15	2.991 (3)	167

Symmetry codes: (i) −*x*, −*y*+1, −*z*+3.
